# Architectural health indicators and the Building Information Model (BIM): Are they relevant to mental health?

**DOI:** 10.1192/j.eurpsy.2023.2099

**Published:** 2023-07-19

**Authors:** E. Abdelmoula, B. Abdelmoula, N. Bouayed Abdelmoula

**Affiliations:** 1 2M2RCA, ENAU Ecole Doctorale Sciences et Ingénierie Architecturales (ED-SIA), Tunis; 2 Genomics of Signalopathies at the service of Medicine, Medical University of Sfax, Sfax, Tunisia

## Abstract

**Introduction:**

BIM for Building Information Modeling is a process that ensures the planning, design, and construction of buildings in an efficient collaborative manner. BIM software encompasses computer files, which can be extracted, exchanged or networked to support decision-making regarding a built asset. It provides physical and functional/semantic digital data representations for building components as a single point of accuracy for all system users. As the design of the built environment plays an important role as a determinant of health, architectural health indicators provide quantitative and empirical data upon which all operators such as architects, customers, BIM users and other stakeholders (public health advisors, construction professionals, healthcare providers, social prescribers, etc.) might monitor and assess the healthiness of architectural design.

**Objectives:**

The objective of this research is to explore the current state of knowledge about architectural health indicators for use in BIM models that address mental health and diseases.

**Methods:**

We comprehensively reviewed the scientific literature using PubMed and Google Scholar as well as electronic bibliographic databases to assess architectural health indicators currently in use by the BIM process, to explore their potential usage and to state the value of indicators focusing on factors affecting mental and social health.

**Results:**

Our bibliographic review revealed that used architectural health indicators in BIM computer systems are very limited. Most of them addressed communicable diseases through simple measurements e.g., air and water quality, etc. However, there is a gap in architectural health indicators pointing non-communicable diseases and their poor health outcomes. Very few indicators focusing on factors affecting mental and social health have been considered in scientific literature.

**Conclusions:**

The research reveals serious gaps in architectural health indicators that address mental health. As there is worldwide a decline of the mental health and given the increase in mental and social health problems, there is an urgent need to address this situation through the incorporation of mental health data, mental disorders and mental disabilities data to enrich the health information of the BIM models and provide an efficient decision support.

**Disclosure of Interest:**

None Declared

